# Trends and predictions of maternal sepsis and other maternal infections among women of childbearing age: a systematic analysis for the global burden of disease study 2019

**DOI:** 10.3389/fpubh.2024.1428271

**Published:** 2024-10-23

**Authors:** Hang Qian, Weifeng Shang, Sheng Zhang, Xiaojun Pan, Sisi Huang, Hui Li, Zhenliang Wen, Jiao Liu, Dechang Chen

**Affiliations:** Department of Critical Care Medicine, Ruijin Hospital, Shanghai Jiao Tong University School of Medicine, Shanghai, China

**Keywords:** maternal sepsis and other maternal infections, global burden of disease, incidence, deaths, age-period-cohort model, prediction

## Abstract

**Background:**

Maternal sepsis and other maternal infections (MSMIs) are major public health concerns worldwide. However, comprehensive data on their global burden and evolving trends remain sparse. This study aims to explore the epidemiological trends of MSMIs in women of childbearing age (WCBA) from 1990 to 2019, investigate the relationship between disease burden and age, period, and birth cohorts, and then provide a prediction of MSMIs incidence and deaths.

**Methods:**

The estimates and 95% uncertainty intervals (UIs) for the incidence and death number of MSMIs in seven age groups (15–19, 20–24, 25–29, 30–34, 35–39, 40–44, 45–49 years) were obtained from the Global Burden of Diseases, Injuries, and Risk Factors Study (GBD) 2019. The age-standardized incidence and mortality rates of MSMIs in WCBA were estimated utilizing the age standardization by direct method. Joinpoint regression analysis was employed to investigate the changing trends of age-standardized incidence and mortality rates from 1990 to 2019. Age-period-cohort analysis was utilized to estimate the independent effects of age, period, and birth cohorts. Furthermore, a Nordpred age-period-cohort analysis was implemented to predict the global epidemiological trends through 2044.

**Results:**

In 2019, the estimated global age-standardized incidence and mortality rates of MSMIs in WCBA were 1072.90 (95% UI: 725.93 to 1483.46) and 0.86 (95% UI: 0.69 to 1.05), respectively. The highest disease burden existed in the African Region. From 1990 to 2019, the estimated global age-standardized incidence and mortality rates of MSMIs (AAPC: -1.32, 95% CI: −1.34 to −1.30; AAPC: -3.39, 95% CI: −4.28 to −2.48) in WCBA both demonstrated significant declining trends. The changing trends varied significantly across 6 regions and 204 countries. The effects of age, period, and cohort on incidence and mortality rates differed. From 2020 to 2044, the global age-standardized incidence rate of MSMIs in WCBA was predicted to decrease whereas the case number increases slowly.

**Conclusion:**

The global trends in MSMIs incidence and mortality generally showed a decline with considerable heterogeneity, indicating both the effectiveness and unevenness of global management of MSMIs. Moreover, the predicted increased case number highlights prominent challenges in the control of MSMIs.

## 1 Introduction

Maternal sepsis and other maternal infections (MSMIs) encompass maternal sepsis during or following labor and delivery as well as other maternal infections believed to have a close epidemiological relationship with pregnancy, including genitourinary tract infections (excluding sexually transmitted diseases), obstetrical wound infections, and breast infections related to childbirth and lactation, which seriously endanger maternal and neonatal health (1–3). On one hand, maternal infections can lead to chronic pelvic inflammatory disease, ectopic pregnancies, infertility, or even death. Specifically, maternal sepsis is one of the primary causes of mortality resulting from such infections. Global estimates suggested that maternal sepsis accounts for approximately 10.7% of maternal deaths globally (1). On the other hand, maternal infections contribute to short-term neonatal conditions like intraventricular hemorrhage (IVH), respiratory distress syndrome (RDS), and necrotizing enterocolitis (NEC); they also cause long-term morbidities such as cerebral palsy and mental retardation, along with preterm births and fetal growth restrictions. Epidemiological studies indicated that there were approximately 1 million neonatal deaths annually attributed to maternal infection or sepsis (4). Although some studies have provided epidemiological data on maternal sepsis and other infectious diseases, most of them are confined to specific geographic areas, lacking age-standardization procedures, which restricts cross-regional and international comparisons (5–8). At the same time, due to the interplay among age, period, and cohort effects, the individual impact of age, period, and cohort effects on incidence and mortality risks remains unclear. Furthermore, it is crucial to predict the incidence and mortality trends of MSMIs, which is vital for optimal medical resource allocation, crafting targeted prevention strategies, and executing effective clinical treatment measures.

The Global Burden of Diseases, Injuries, and Risk Factors Study (GBD) 2019 offers a standardized and comprehensive assessment of the global burden of 369 diseases, injuries, and impairments and 87 risk factors across 204 countries and territories, providing a critical resource for epidemiological research (9). Data from GBD 2019 have been instrumental in various analytical frameworks, including disease burden descriptions, trend analyses, future projections, and health inequality monitoring, thus offering crucial insights for public policy formulation and health promotion initiatives (10–13). In our study, we utilized data from GBD 2019 to calculate the age-standardized incidence and mortality rates of MSMIs among women of childbearing age (WCBA), explore the changes from 1990 to 2019, assess the impact of age, period, and birth cohorts, and predict the incidence and mortality rates over the next 25 years. We aim to provide a comprehensive perspective to inform the development of healthcare policies for the prevention and control of MSMIs at global, regional, and national levels.

## 2 Materials and methods

### 2.1 Study population

WCBA exhibits robust fertility marked by periodic fluctuations in sex hormones. The World Health Organization (WHO) categorized women aged 15 to 49 as WCBA (14).

### 2.2 Data sources

The estimates and 95% uncertainty intervals (UIs) for incidence and death number of MSMIs in seven age groups (15–19, 20–24, 25–29, 30–34, 35–39, 40–44, 45–49 years) were obtained from the GBD 2019, which offers a comprehensive scientific examination of published, publicly available, and contributed data on disease and injury incidence, prevalence, and mortality for 369 diseases, injuries, and impairments and 87 risk factors across 204 countries and territories (9). Age-standardized incidence rate (ASIR) and age-standardized mortality rate (ASDR) of MSMIs in WCBA were calculated using the direct method, which presumes that the rates are distributed as a weighted sum of independent Poisson random variables (15). Both ASIR and ASDR were presented as the estimate per 100,000 population, along with their 95% UI.

### 2.3 Temporal trend analysis

The Joinpoint regression model is a group of linear statistical models that avoids the subjectivity of conventional trend studies based on linear trends by estimating the changing rule of sickness rates using the least squares approach (16). The rates in the model were logarithmically transformed, and standard errors were determined using binomial approximation (16). It enables the identification of the points with notable changes in trend, segmentation of longitudinal variations via segmented regression, and evaluation of segment trends through the calculation of the annual percentage change (APC) and the 95% confidence interval (CI) (17). Furthermore, the average APC (AAPC), acting as a comprehensive indicator of the trend across a specified fixed interval, is determined as a weighted average of APC over the segmented intervals’ durations. An increasing trend is indicated when both the AAPC estimate and its 95% CI lower boundary >0. Conversely, a decreasing trend is suggested when the AAPC estimate and its 95% CI upper boundary are both <0.

### 2.4 Age-period-cohort analysis

Age-period-cohort (APC) model is a statistical method that has been known in biological, health, and social sciences for decades (18). Based on Poisson distributions, the APC model effectively captures temporal trends in incidence or mortality by age, period, and cohort (19). Specifically, the APC model with intrinsic estimator (IE) method is capable of decomposing three temporal trends and provides unbiased and relatively efficient estimation results (20).

The APC-IE method presents estimated coefficients for the age, period, and cohort effects, and then relative risk (RR) = exp. (effect coefficient) was calculated. A RR value of greater than 1 signifies a higher risk of incidence or mortality relative to each average level. Conversely, a RR value of less than 1 indicates a lower risk than average (21).

When preparing data for the APC-IE model, the incidence and mortality of MSMIs and population data are arranged into seven 5-year distinct age groups (15–19, 20–24, 25–29, 30–34, 35–39, 40–44, 45–49 years). Correspondingly, the period from 1990 to 2019 was divided into six 5-year periods (1990–1994, 1995–1999, 2000–2004, 2005–2009, 2010–2014, 2015–2019). Thus, 12 consecutive 5-year birth cohort groups were also created (1945–1949, 1950–1954, 1955–1959, 1960–1964, 1965–1969, 1970–1974, 1975–1979, 1980–1984, 1985–1989, 1990–1994, 1995–1999, 2000–2004). Later, Stata 16.0 was used to perform the APC APC-IE model.

### 2.5 Prediction analysis

To reflect the trends of MSMIs burden in the next 25 years, the case number and age-standardized incidence and mortality rates of MSMIs in WCBA were predicted utilizing the Nordpred package (in R software). The package takes into account changing rates and changing population structures, which has demonstrated good performance in forecasting future trends in disease incidence and mortality (22, 23).

The statistical procedures were conducted using the R program (version 4.3.2, R core team). *p* < 0.05 was considered statistically significant difference.

## 3 Results

### 3.1 Trends of MSMIs incidence and mortality in WCBA

**Figure 1:**
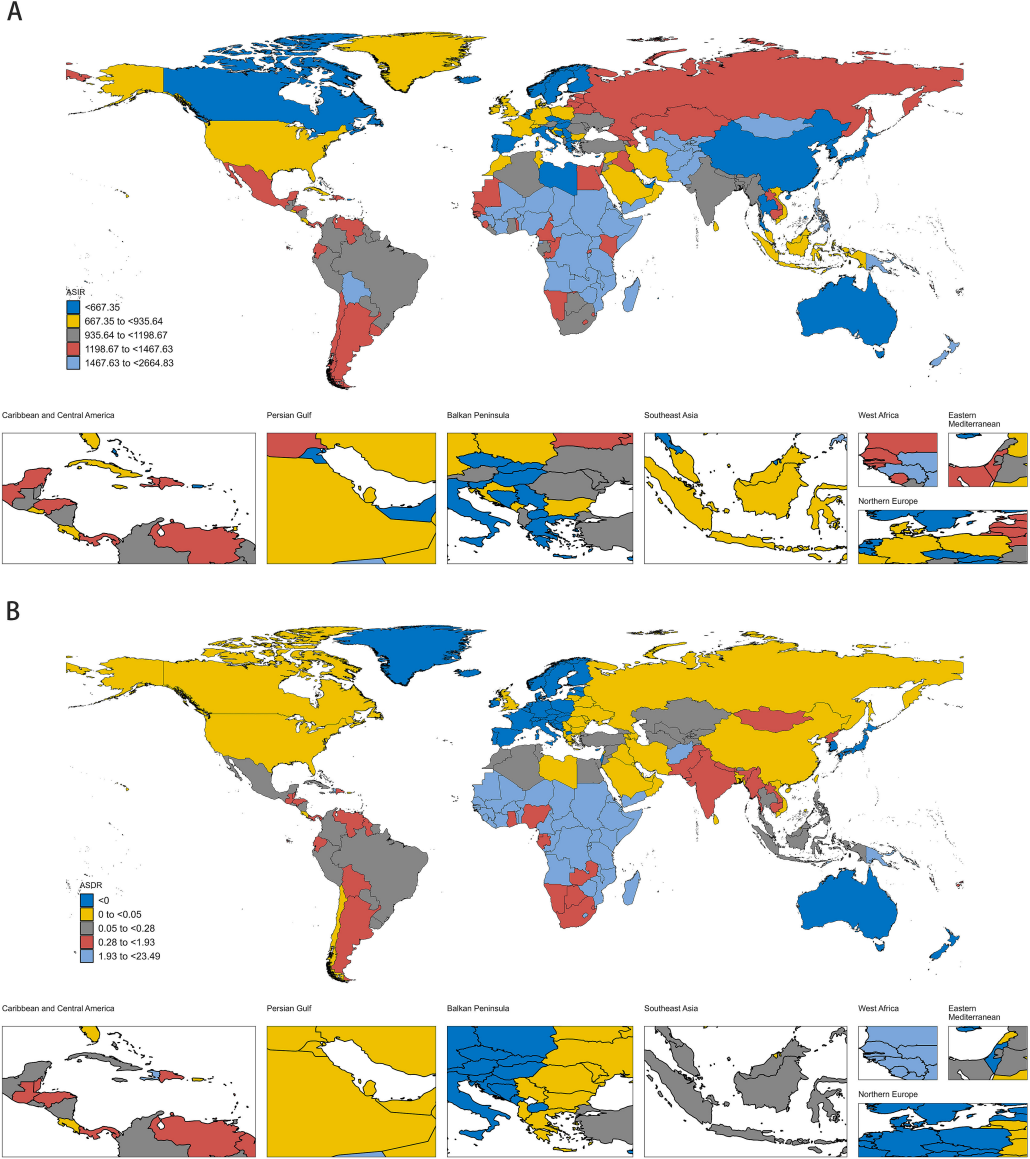
The ASIR (A) and ASDR (B) of MSMIs in WCBA in 204 countries/territories in 2019. ASIR, age-standardized incidence rate; ASDR, age-standardized death rate; MSMIs, maternal sepsis and other maternal infections; WCBA, women of childbearing age.

**Figure 2:**
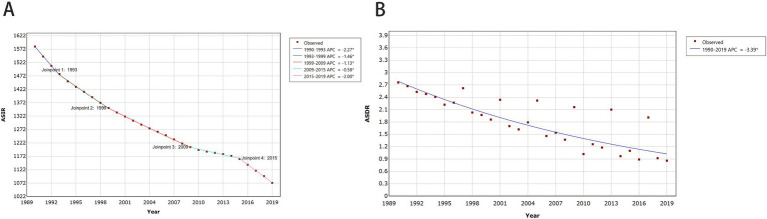
Global trends for ASIR (A) and ASDR (B) of MSMIs in WCBA from 1990 to 2019. APC, annual percentage change; ASIR, age-standardized incidence rate; ASDR, age-standardized death rate; MSMIs, maternal sepsis and other maternal infections; WCBA, women of childbearing age.

In 2019, the estimated global age-standardized incidence and mortality rates of MSMIs in WCBA were 1072.90 (95% UI: 725.93 to 1483.46) and 0.86 (95% UI: 0.69 to 1.05), respectively, with the highest age-standardized incidence and mortality rates in African Region (Figure 1; Supplementary Tables S1, S2). At the national level, the estimated age-standardized incidence and mortality rates showed significant variation across 204 countries and territories, with the highest age-standardized incidence and mortality rates in Somalia, and the lowest age-standardized incidence and mortality rates in Singapore and Iceland, respectively (Figure 1; Supplementary Tables S1, S2). All these indicated an imbalance in the distribution of disease burden. From 1990 to 2019, the estimated global age-standardized incidence of MSMIs in WCBA displayed a significant decreasing trend (AAPC = −1.32, 95% CI: −1.34 to −1.30; *p* < 0.001), with the most substantial changes at 1995–2014 (APC = −2.27, 95% CI: −2.40 to −2.15; *p* < 0.001; Figure 2A; Supplementary Table S3). Similarly, the estimated global ASDR of MSMIs in WCBA also indicated a significant decline (AAPC = −3.39, 95% CI: −4.28 to −2.48; *p* < 0.001; Figure 2B; Supplementary Table S3). At the regional level, the estimated age-standardized incidence and mortality rates declined across all regions, with the most significant decrease in age-standardized incidence and mortality rates in the South-East Asia Region (Supplementary Table S3). At the national level, the trends of estimated age-standardized incidence and mortality rates showed significant variation across 204 countries and territories, with the most significant decrease in age-standardized incidence and mortality rates occurring in Cyprus, Bosnia and Herzegovina, respectively (Supplementary Table S3), these suggested the efficacy of disease management over the past three decades. In addition, the AAPCs in global MSMIs incidence and death by different age groups from 1990 to 2019 are demonstrated in Table 1. Both ASIR and ASDR showed downward trends across all age groups (Table 1; Supplementary Figures S1, S2). Compared to the older age groups, the younger age groups exhibited more substantial declines in incidence and mortality (Table 1; Supplementary Figures S1, S2), suggesting that age appears to be one of the factors influencing the trend of changes in disease burden.

**Table 1:** The global AAPCs in ASIR and ASDR of MSMIs in WCBA from 1990 to 2019 by age group.

Age group (year)	Incidence (ASIR)			Mortality (ASDR)		
	AAPC	95% CI	*p*-value	AAPC	95% CI	*p*- value
15–19	−1.68*	(−1.77,-1.60)	<0.001	−3.95*	(−4.44,-3.46)	<0.001
20–24	−1.64*	(−1.67,-1.61)	<0.001	−4.24*	(−4.50,-3.57)	<0.001
25–29	−1.12*	(−1.18,-1.07)	<0.001	−4.09*	(−4.40,-3.97)	<0.001
30–34	−0.67*	(−0.71,-0.63)	<0.001	−4.05*	(−4.39,-3.72)	<0.001
35–39	−0.58*	(−0.64,-0.52)	<0.001	−3.64*	(−3.84,-3.44)	<0.001
40–44	−1.20*	(−1.31,-1.08)	<0.001	−3.24*	(−3.41,-3.08)	<0.001
45–49	−2.33*	(−2.48,-2.18)	<0.001	−3.40*	(−4.14,-2.65)	<0.001

AAPC, average annual percentage change; CI, confidence interval; ASIR, age-standardized incidence rate; ASDR, age-standardized death rate; MSMIs, maternal sepsis and other maternal infections; WCBA, women of childbearing age.

*Significantly different from 0 at alpha = 0.05 (*p* < 0.05).

### 3.2 Age-period-cohort effects on MSMIs incidence and mortality in WCBA

**Figure 3:**
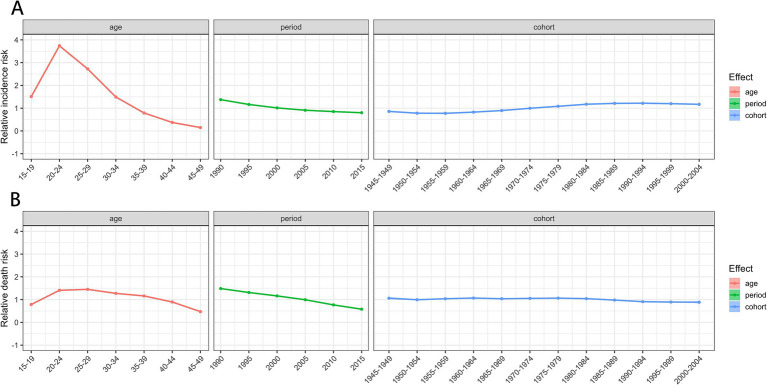
The relative risk of MSMIs incidence (A) and deaths (B) in WCBA from 1990 to 2019. MSMIs, maternal sepsis and other maternal infections; WCBA, women of childbearing age.

Trends in the age, period, and cohort-specific incidence and mortality rates of MSMIs in WCBA are presented in Supplementary Figures S3, S4. The incidence and mortality rates of MSMIs in all periods and cohorts increased over age and peaked at ages 20–24, and subsequently declined, reaching their lowest levels at ages 45–49. Newer periods and cohorts have lower incidence and mortality rates in most of the age groups. The distribution by period according to cohorts did not show significant variation. Cohorts from 1970 to 1999 had the highest incidence and mortality rates and dropped fast afterward across different periods (Supplementary Figures S3, S4). To sum up, there is an interactive effect of age, period, and cohort factors on the risks of incidence and mortality. For the incidence risk of MSMIs in WCBA, the age effect revealed sharp increasing trends from 15 to 24 years, followed by a significant decline through 49 years (Table 2; Figure 3A), revealing the incidence patterns across different age groups. The period effect demonstrated a consistent decline with the advancing period, indicating that the occurrence of MSMIs in WCBA had been effectively controlled to some extent over time. Seemingly, incidence risk substantially decreased from 1990 (RR = 1.3748; 95% CI 1.3739, 1.3757) to 2015 (RR = 0.7997; 95% CI 0.7991, 0.8002; Table 2; Figure 3A). Regarding the cohort effect, an increased incidence risk was observed from the 1955–1959 to the 1990–1994 birth cohorts, followed by a slight decrease up to the 2000–2004 birth c For the mortality risk of MSMIs in WCBA, the age effect showed an increasing trend from 15 to 29 years, followed by a gradual decline until 49 years (Table 2; Figure 3B). The period effect consistently diminished over successive periods (Table 2; Figure 3B). Seemingly, mortality risk substantially decreased from 1990 (RR = 1.4832; 95% CI 1.4703, 1.5003) to 2015 [RR = 0.5789; 95% CI 0.5704, 0.5 cohort (Table 2; Figure 3A; 0.875)] (Table 2; Figure 3B). The cohort effect demonstrated a gradual decline from 1975 to 1979 to 2000–2004 birth cohort (Table 2; Figure 3B).

**Table 2:** The global incidence and mortality relative risks of MSMIs in WCBA due to age, period, and cohort effects.

Factor	Incidence			Mortality		
	RR	95%CI	*P*-value	RR	95%CI	*P*-value
Age(years)						
15–19	1.5090	1.5076–1.5100	0.0000	0.7852	0.7746–0.7958	0.0000
20–24	3.7404	3.7476–3.7423	0.0000	1.4080	1.3933–1.4228	0.0000
25–29	2.7253	2.7276–2.7266	0.0000	1.4511	1.4359–1.4666	0.0000
30–34	1.4919	1.4976–1.4928	0.0000	1.2733	1.2587–1.2880	0.0000
35–39	0.7890	0.7876–0.7897	0.0000	1.1609	1.1464–1.1756	0.0000
40–44	0.3728	0.3776–0.3733	0.0000	0.8956	0.8828–0.9087	0.0000
45–49	0.1481	0.1476–0.1484	0.0000	0.4709	0.4617–0.4802	0.0000
Period						
1990	1.3748	1.3739–1.3757	0.0000	1.4852	1.4703–1.5003	0.0000
1995	1.1630	1.1624–1.1636	0.0000	1.3126	1.2989–1.3265	0.0000
2000	1.0118	1.0114–1.0122	0.0000	1.1618	1.1497–1.1739	0.0000
2005	0.9093	0.9089–0.9097	0.0000	0.9939	0.9832–1.0046	0.2620
2010	0.8501	0.8497–0.8505	0.0000	0.7674	0.7584–0.7765	0.0000
2015	0.7997	0.7991–0.8002	0.0000	0.5789	0.5704–0.5875	0.0000
Cohort						
1945–1949	0.8574	0.8540–0.8608	0.0000	1.0610	1.0166–1.1074	0.0070
1950–1954	0.7799	0.7779–0.7818	0.0000	0.9946	0.9673–1.0228	0.7050
1955–1959	0.7738	0.7724–0.7752	0.0000	1.0372	1.0146–1.0603	0.0010
1960–1964	0.8264	0.8252–0.8276	0.0000	1.0688	1.0488–1.0892	0.0000
1965–1969	0.8960	0.8951–0.8970	0.0000	1.0374	1.0206–1.0545	0.0000
1970–1974	0.9957	0.9948–0.9965	0.0000	1.0527	1.0377–1.0679	0.0000
1975–1979	1.0820	1.0813–1.0827	0.0000	1.0645	1.0504–1.0787	0.0000
1980–1984	1.1730	1.1724–1.1736	0.0000	1.0421	1.0277–1.0568	0.0000
1985–1989	1.2111	1.2105–1.2116	0.0000	0.9804	0.9659–0.9951	0.0090
1990–1994	1.2170	1.2164–1.2177	0.0000	0.9097	0.8942–0.9256	0.0000
1995–1999	1.1985	1.1977–1.1994	0.0000	0.8937	0.8740–0.9138	0.0000
2000–2004	1.1693	1.1677–1.1709	0.0000	0.8852	0.8459–0.9264	0.0000

RR, relative risk [RR ¼ exp(coefficient)]; CI, confidence interval; MSMIs, maternal sepsis and other maternal infections; WCBA, women of childbearing age.

### 3.3 Predictions of MSMIs incidence and mortality in WCBA for 2044

**Figure 4:**
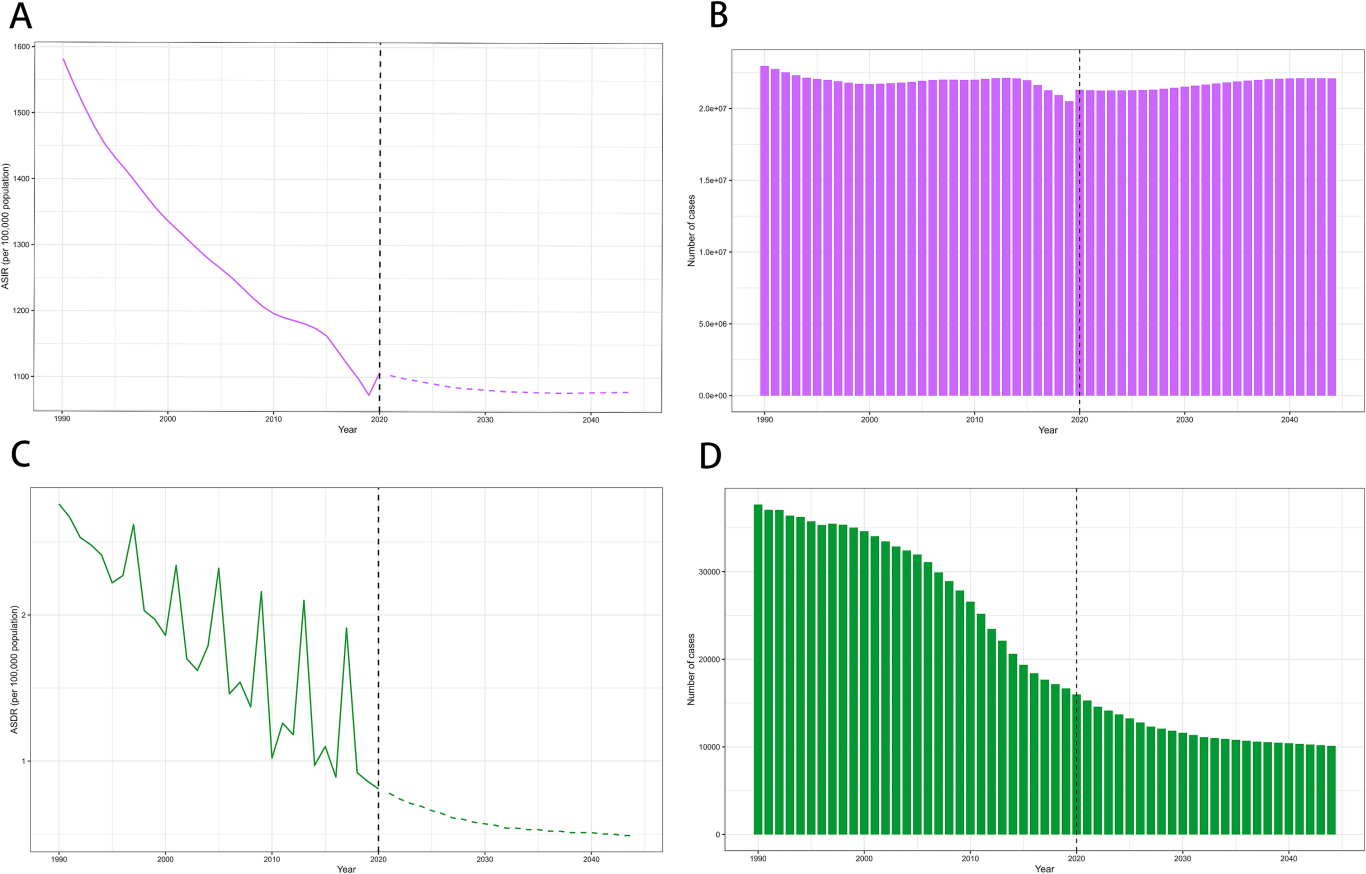
The global trends in ASIR and case number of incidence (A,B), ASDR and case number of mortality (C,D) of MSMIs in WCBA from 1990 to 2044. ASIR, age-standardized incidence rate; ASDR, age-standardized death rate; MSMIs, maternal sepsis and other maternal infections; WCBA, women of childbearing age. *The observed rates are plotted with solid lines, and predicted rates are plotted with dashed lines.

The global ASIR and ASDR of MSMIs in WCBA are predicted to keep on decreasing in the next 25 years (Figure 4). The number of deaths of MSMIs is also predicted to continue to decrease globally, from 16,670 in 2019 to 10,123 in 2044. However, the number of incident cases of MSMIs is predicted to slowly increase globally, from 20,503,385 in 2019 to 22,095,012 in 2044.

## 4 Discussion

In this study, we employed data from GBD 2019 to calculate the age-standardized incidence and mortality rates of MSMIs in WCBA from 1990 to 2019 and examine their changing trends at the global, regional (African Region, Eastern Mediterranean Region, European Region, Region of the Americas, South-East Asia Region, Western Pacific Region), and national (204 countries and territories) levels. Given the complex interplay among age, period, and cohort effects, we explored the relative impact of these variables on the incidence and mortality trends of MSMIs. Additionally, we predicted the global number of cases and rates of incidence and mortality for the next 25 years (2019–2044).

The United Nations proposed the Targets of Sustainable Development Goal 3 in 2015, with the goal of reducing maternal mortality to fewer than 70/100,000 live births globally by 2030 (24). The control of MSMIs is closely linked to the achievement of this target to reduce maternal mortality, as infections, especially sepsis, are a significant and undeniable cause of maternal deaths. Relevant data indicated that infections are responsible for approximately 11% of maternal deaths and contribute to many deaths attributed to other conditions (1). Furthermore, in 2017, the WHO called for an acceleration in the reduction of preventable maternal and neonatal deaths related to sepsis by 2030 (2). Undoubtedly, gaining a deeper understanding of the epidemiological characteristics of MSMIs in WCBA can aid in assessing the potential for achieving related targets and assist in the allocation of public health and medical resources.

In 2019, there were notable variations in the ASIR and ASDR of MSMIs in WCBA at global, regional, and national levels. This suggests substantial heterogeneity in the disease’s burden across different areas, potentially attributed to various factors such as genetic variations and environmental exposures (4). Particularly noteworthy is the highest age-standardized incidence and mortality rates of MSMIs in WCBA occurred in the African region at the regional level. Correspondingly, the highest age-standardized incidence and mortality rates at the national level occurred in Somalia in Africa. While the lowest age-standardized incidence and mortality rates occurred in Singapore and Iceland, respectively. Several potential reasons might be attributable to explain this phenomenon. Firstly, Somalia has a lower level of economic development than Singapore and Iceland. There seems to be a concentration of disease burden in relatively underdeveloped areas, aligning with the findings of previous studies (25–29). A prospective cohort study of 2,850 hospitalized pregnant or recently pregnant women with suspected or confirmed infections revealed that the distribution of severity in maternal outcomes varies by income level across countries (29). Notably, low-income countries recorded the highest rates of women experiencing severe maternal outcomes related to infections (29). Chen et al. conducted an analysis based on GBD 2019 data indicating a negative correlation between the Socio-Demographic Index (SDI) levels and the incidence and mortality rates of MSMIs (26). Generally, a higher SDI level is associated with a higher quality healthcare system and superior medical services, thereby reducing the disease burden. Secondly, ethnicity significantly influences the development of MSMIs, with research indicating that Black and other ethnic minority groups face nearly double the risk of maternal sepsis (4, 7, 30). Thirdly, ineffective governance in Somalia, war conflicts, and climate shocks are also important factors (31).

From 1990 to 2019, the global age-standardized incidence and mortality rates of MSMIs in WCBA showed a significant declining trend, consistent with previous studies (26). This indicates substantial progress in the management and treatment of these conditions over the past few decades. At the regional level, the estimated age-standardized incidence and mortality rates declined across all regions, with the most significant decrease occurring in the South-East Asia Region. At the national level, significant variations in the trends of estimated age-standardized incidence and mortality rates across 204 countries highlight considerable disparities in the prevention, management, and treatment of MSMIs in WCBA globally. In addition, the high heterogeneity of the disease, along with differences in the genetic ethnicities of regional populations and environmental variations could also account for these observed distribution differences. Identifying each region or country’s unique trend is crucial for guiding governments in improving their healthcare systems to meet the diverse medical needs related to MSMIs in WCBA and underscores the necessity of equitably allocating medical resources related to the prevention and treatment of MSMIs globally. In summary, the overall decline in the incidence and mortality rates is encouraging for MSMIs patients and their families globally. For policymakers, there is an urgent need to ready healthcare resources for the increasing risk of maternal individuals suffering from MSMIs in the relatively low SDI regions. In clinical practice, it is necessary to strengthen the training of medical staff to enhance their focus on maternal health. Moreover, WCBA is encouraged to place great importance on the treatment of maternal infections.

The age effects explain how the interest indicators for a disease change with age, reflecting the inherent nature of age changes (32). Our study indicated that age had an impact on the incidence and mortality rates of MSMIs. The global high-risk age groups for the incidence and mortality of MSMIs are 20–24 years and 25–29 years, respectively. Previous studies have reported similar incidence patterns of MSMIs, with a lack of data on mortality rates (6). In these age groups, women are at their peak fertility, accompanied by a decline in immune function, making them highly susceptible to infections (33). Moreover, women in these stages often lack adequate knowledge of prenatal and postnatal care, and the insufficient social support systems may result in early infections not being promptly identified and treated, potentially progressing to sepsis. This underscores the importance of providing special attention and health management for these age groups. The period effect on the incidence and mortality of MSMIs markedly decreased globally, where advancements in healthcare may play an important role. Over recent decades, research related to MSMIs has seen significant improvements in both quantity and quality. The outcomes of high-quality basic and clinical research have continuously advanced the diagnosis and treatment of sepsis. Currently, the treatment of MSMIs primarily involves organ support therapy and the appropriate antibiotics management (27). The widespread adoption of multidisciplinary teams (MDTs) for the early detection and management of sepsis in pregnant women aids in improving patient outcomes. The cohort effect indicates that different birth cohorts have an impact on the incidence and mortality rates of MSMIs. Variations in the RR of incidence and mortality may indicate changes in exposure to environmental risk factors or lifestyle changes within the same birth cohort. Significantly, the RR of MSMIs incidence has partially increased in the 1955–1994 birth cohorts, implying a rise in exposure to risk factors for MSMIs. The cohort effect on the mortality of MSMIs exhibited a decreasing trend from earlier birth cohorts to more recent birth cohorts. A probable explanation is that more recent birth cohorts tend to have higher levels of education, a greater awareness of health and illness, and are more proactive in treatment compared with earlier birth cohorts (34). At the same time, earlier-born cohorts might have undergone wars, which not only worsened living and hygiene conditions but also directly threatened life and health. Additionally, advancements in medical technology and the emergence of new diagnostic and treatment methods have also contributed to the decrease in RR of MSMIs mortality.

From 2020 to 2044, the age-standardized incidence and mortality rates, as well as the number of deaths, are showing a downward trend. This improvement can be attributed to better diagnostic and treatment measures that enhance patient outcomes. At the same time, improved health education for pregnant women could also help reduce the future disease burden of MSMIs. Furthermore, a related study forecasted that fertility rates will continue to decline worldwide from a global total fertility rate (TFR) of 2.21(95% UI 2.06–2.36) in 2022 to 1.83 (1.59–2.08) in 2050, which could also be attributable to reduce the future disease burden of MSMIs (35). It is noteworthy that the global case number of MSMIs in WCBA was predicted to increase, which might be related to global population growth. The United Nations has highlighted that the global population is on a continuous rise, expected to reach 8.5 billion in 2030 and 9.7 billion in 2050 (36). Specifically, the number of WCBA worldwide is projected to increase from 2 billion in 2024 to about 2.2 billion in 2050 (37). Thus, governments should take into account the potential health impacts of population growth when devising or modifying health prevention strategies.

This study has the following strengths. Firstly, our study offered a deeper insight into the temporal trends of MSMIs, thereby providing valuable perspectives for epidemiology and the formulation of health policies. Specifically, differentiating the age-period-cohort effects allows us to identify the primary factors driving changes. Secondly, the estimates of age-standardized incidence and mortality rates of MSMIs in WCBA facilitate effective comparisons between different regions and provide a data foundation for further scientific research. Lastly, the prediction of incidence and mortality trends for the next 25 years is significant for the protection and promotion of public health.

However, there are also several limitations. Firstly, the imperfection of healthcare systems in underdeveloped countries may lead to underdiagnosis, misdiagnosis, and consequently, the underestimation or overestimation of disease burden. Secondly, to address the variable quality stemming from the vast quantities of raw data collected from various countries, GBD collaborators employed thorough data cleaning processes and extensive advanced statistical modeling. However, this approach might lead to a significant dependence on modeled data within the GBD dataset. Thirdly, the types of MSMIs included in the GBD 2019 are limited, which may restrict our ability to conduct a broad exploration. Fourthly, due to a lack of detailed data, it is not possible to explore the characteristics of epidemiological trends for MSMIs at the sub-national level. Lastly, there is a certain degree of latency in GBD data. Future analyses are required to incorporate the latest GBD database for further exploration.

## 5 Conclusion

Our research presented significant disparities in the burden of MSMIs in WCBA, suggesting that governments worldwide should develop flexible health policies, allocate medical resources wisely, and improve healthcare systems. Additionally, we predicted that the case number of MSMIs will increase by 2044, highlighting the substantial challenges faced in disease control. More customized prevention strategies and medical resources for maternal health are required to be established in the future.

## Data Availability

The original contributions presented in the study are included in the article/Supplementary material, further inquiries can be directed to the corresponding authors.

## Supplementary material

Supplementary Figure S1 Global trends for ASIR (A) and ASDR (B) of MSMIs in WCBA from 1990 to 2 age group. APC, annual percentage change; ASIR, age-standardized incidence rate; ASDR, standardized death rate; MSMIs, maternal sepsis and other maternal infections; WCBA, women of childbearing age.

Supplementary Figure S2 Global trends for ASIR (A) and ASDR (B) of MSMIs in WCBA from 1990 to 2019 by age group. APC, annual percentage change; ASIR, age-standardized incidence rate; ASDR, age- standardized death rate; MSMIs, maternal sepsis and other maternal infections; WCBA, women of childbearing age.

Supplementary Figure S3 Age-period-cohort-specific incidence rates of MSMIs in WCBA from 1990 to 2019. (A) Age-specific incidence rate grouped by period; (B) Age-specific incidence rate grouped by cohort; (C) cohort-specific incidence rate grouped by period.

Supplementary Figure S4 Age-period-cohort-specific mortality rates of MSMIs in WCBA from 1990 to 2019. (A) Age-specific mortality rate grouped by period; (B) Age-specific mortality rate grouped by cohort; (C) cohort-specific incidence mortality grouped by period.
