# Supplementary figures and images for: Trends and predictions of maternal sepsis and other maternal infections among women of childbearing age: a systematic analysis for the global burden of disease study 2019
Source: Front Public Health. 2024 Oct 23;12:1428271. doi: 10.3389/fpubh.2024.1428271 (PMC11538001; doi:10.3389/fpubh.2024.1428271)

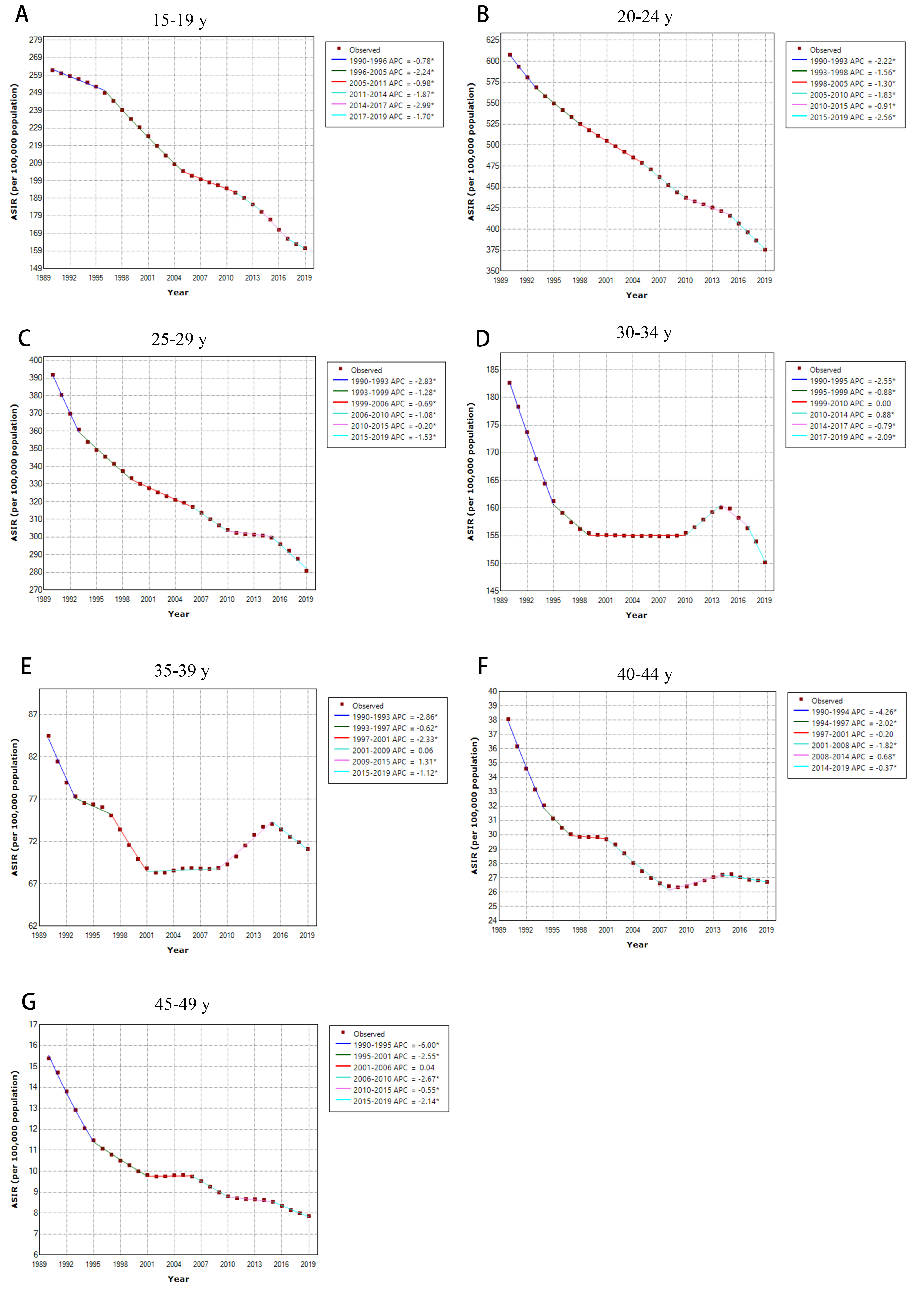

Supplement: Supplementary Figure S1 — Global trends for ASIR (A) and ASDR (B) of MSMIs in WCBA from 1990 to 2 age group. APC, annual percentage change; ASIR, age-standardized incidence rate; ASDR, standardized death rate; MSMIs, maternal sepsis and other maternal infections; WCBA, women of childbearing age. [file Image_1.TIF]

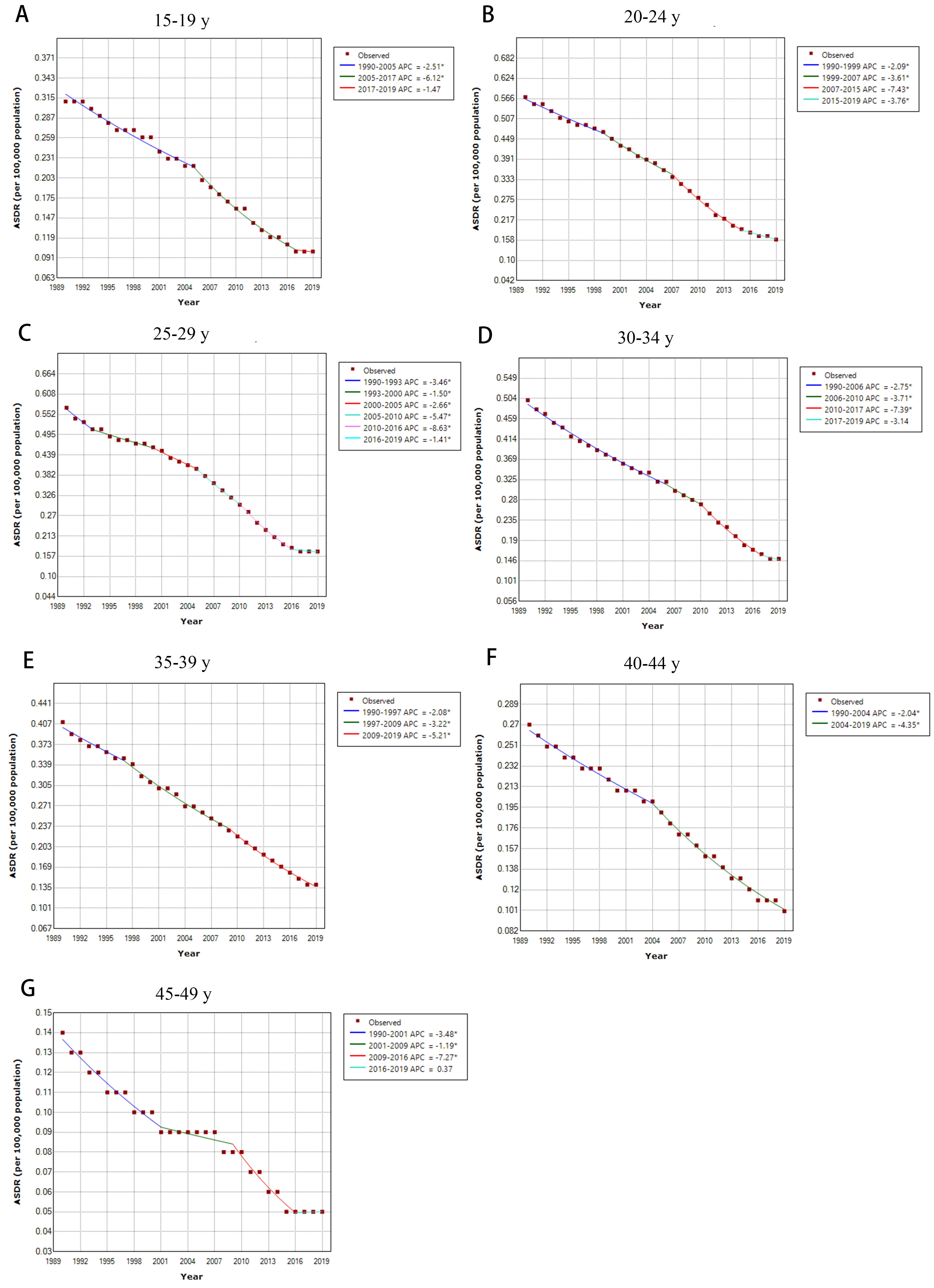

Supplement: Supplementary Figure S2 — Global trends for ASIR (A) and ASDR (B) of MSMIs in WCBA from 1990 to 2019 by age group. APC, annual percentage change; ASIR, age-standardized incidence rate; ASDR, age- standardized death rate; MSMIs, maternal sepsis and other maternal infections; WCBA, women of childbearing age. [file Image_2.TIF]

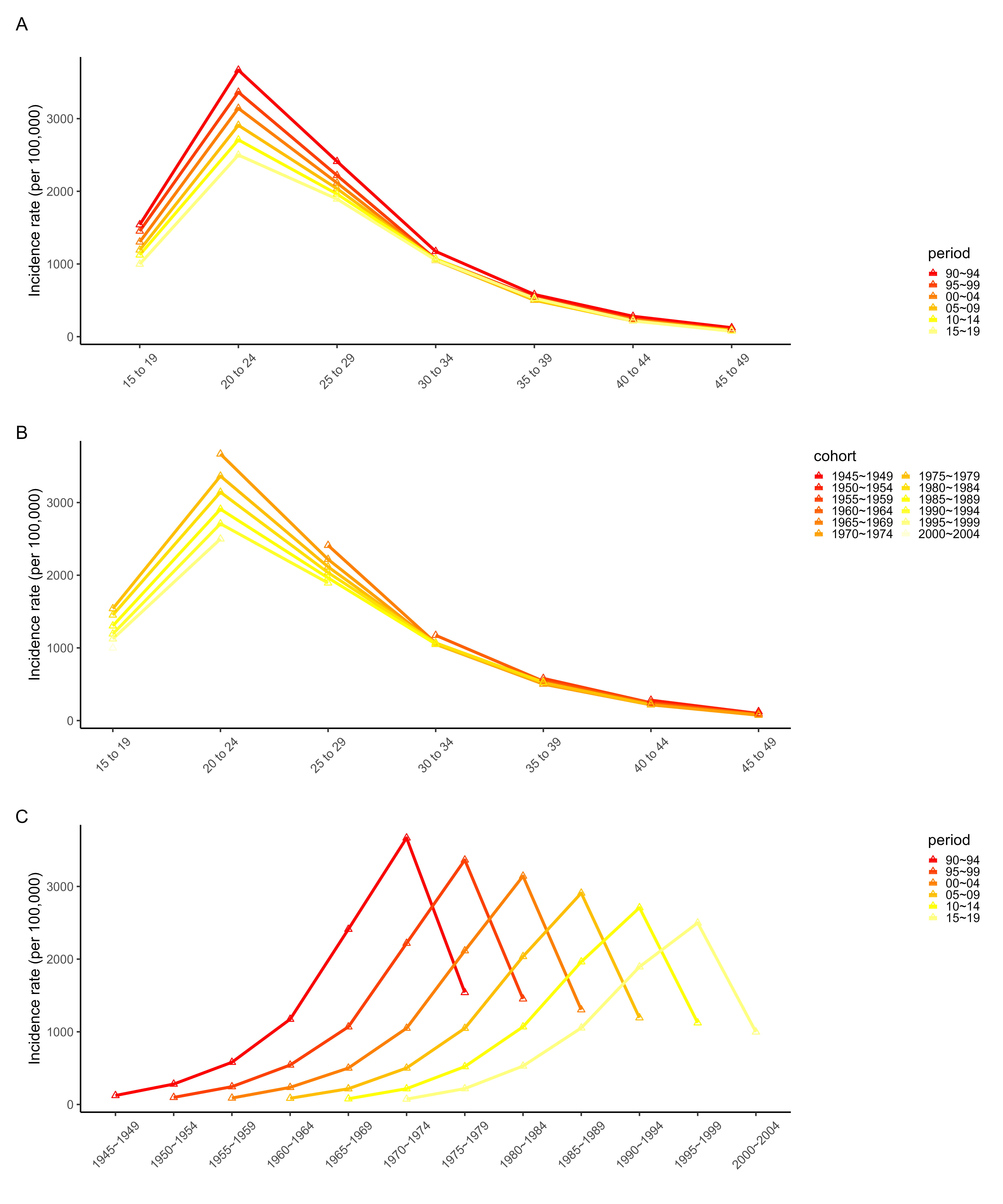

Supplement: Supplementary Figure S3 — Age-period-cohort-specific incidence rates of MSMIs in WCBA from 1990 to 2019. (A) Age-specific incidence rate grouped by period; (B) Age-specific incidence rate grouped by cohort; (C) cohort-specific incidence rate grouped by period. [file Image_3.JPEG]

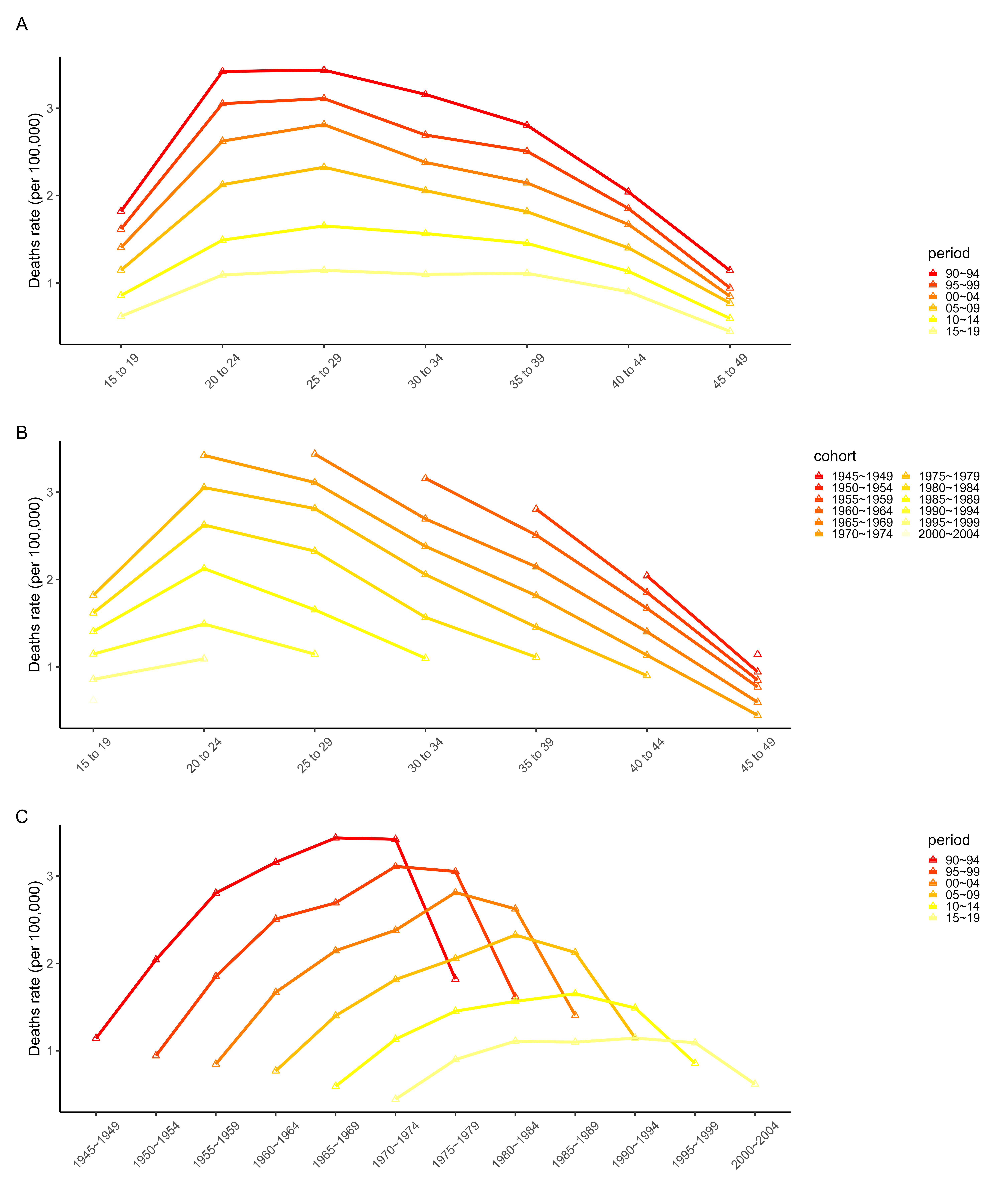

Supplement: Supplementary Figure S4 — Age-period-cohort-specific mortality rates of MSMIs in WCBA from 1990 to 2019. (A) Age-specific mortality rate grouped by period; (B) Age-specific mortality rate grouped by cohort; (C) cohort-specific incidence mortality grouped by period. [file Image_4.JPEG]
